# Simulation study of inverse diffusion counterbalance method for super-resolution ion mobility spectrometry

**DOI:** 10.3389/fchem.2022.1004615

**Published:** 2022-09-21

**Authors:** Kaitai Guo, Yang Zheng, Haihong Hu, Jimin Liang

**Affiliations:** School of Electronic Engineering, Xidian University, Xi’an, Shaanxi, China

**Keywords:** IMS, super-resolution, inverse diffusion counterbalance method, diffusion, diffusion limit resolution

## Abstract

Ion mobility spectrometer (IMS) is a powerful chemical composition analysis tool working at atmospheric pressure that can be used to separate complex samples and study molecular structures. Resolution is a key parameter for evaluating the performance of IMS. However, for the pulsed sampling technique used by drift tube IMS, there is an upper limit to the resolution due to the diffusion between ions and the drift gas. In this work, an inverse diffusion counterbalance method is proposed to break the resolution limit. The method is inspired by the stimulated emission depletion (STED). In optical microscopy systems, STED is used to break the optical diffraction limit by a ring of depleted light to counteract diffraction effects of the excited light. We modified this strategy and applied it to an IMS system for counteracting the diffusion effect of the pulsed ion packet. The method can increase the resolution up to 1.55 times through theoretical analysis, and the improvement is verified by simulations. The simulation results find that the initial width of the ion packet has an influence on the effectiveness of the method, and the narrower the initial width, the better the effect. The proposed inverse counterbalance strategy may also be applied to other spectral analysis instruments to break the resolution limit.

## 1 Introduction

Ion mobility spectrometer (IMS) (Eiceman et al., 2013; Sorribes-Soriano et al., 2018) is an analytical instrument working at atmospheric pressure. It has a wide range of applications in many fields, such as drug detection (Zou et al., 2018), medical diagnosis (Ruszkiewicz et al., 2020), and compound analysis (Han and Yao, 2020; Wu et al., 2021). IMS has many kinds of derivatives, such as Travelling wave ion mobility spectrometry (TW-IMS) (Giles et al., 2004; Pringle et al., 2007), Trapped ion mobility spectrometry (TIMS) (Fernandez-Lima et al., 2011b,a), Differential or field asymmetric ion mobility spectrometry (DMS/FAIMS) (Buryakov et al., 1993, 1991), Fourier transform ion mobility spectrometry (FT-IMS) (Knorr et al., 1985), Hadamard transform ion mobility spectrometry (HT-IMS) (Clowers et al., 2006; Szumlas et al., 2006), etc. The development of special configurations (Michelmann et al., 2014; Hernandez et al., 2014; Silveira et al., 2014) and post-processing technologies (Davis and Clowers, 2018; May et al., 2020) allows the instrument to reach a very high level of resolution. Because the mobility can be obtained directly without calibration (Kirk et al., 2019a), the conventional drift tube ion mobility spectrometry (DT-IMS) still has a wide range of applications. Unless otherwise indicated, all IMS mentioned in the following are DT-IMS. For conventional drift tube IMS, the ionized sample is sent into the drift region as a pulsed ion packet. The drift region is filled with neutral buffer gas, in which ions will drift a certain distance under the influence of electric field. Due to the difference in mobility, packets of different ion species are separated depending on their drift time. The separation capability can be quantified by the resolution of IMS (Rokushika et al., 1985), which is defined as the arrival time of a pulsed ion packet *t*_d_ divided by its full width at half maximum *t*_fwhm_.

Lengthening the drift region while increasing the drift voltage is an effective way to improve the resolution (Siems et al., 1994; Watts and Wilders, 1992; Dugourd et al., 1997; Wu et al., 1998). However, a significant increase in drift length leads to a rather large and heavy device, and some short-lived ions will disappear due to the extended drift time. In recent years, reducing the initial width of the ion packet *w*_i_ has proven to be an effective way to improve the resolution. Since the ion gate is the key component to control the entry of ions, the *w*_i_ can be effectively shortened by making improvements to the ion gate. Du et al. (Du et al., 2012) studied three different effects that occur when a Bradbury-Nielsen gate is running, and used the compression effect to achieve a shortening of *w*_i_. The compression effect was later shown to work also for the field switching gate IMS (Chen et al., 2019) and Tyndall-Powell gate IMS (Chen et al., 2015), both of which give an increase in resolution. Langejuergen et al. (Langejuergen et al., 2014; Reinecke et al., 2016) have proposed for the first time a three-grid shutter structure to control the entry of ions into the drift region. This type of ion gate can use the potential switching on the three metal grids to achieve precise ion control, which can make *w*_i_ much shorter. Combined with a high kinetic energy drift tube, a very high level of resolution can be achieved (Kirk et al., 2018; 2019b).

However, whether increasing the drift length or decreasing the *w*_i_, these methods can only improve the resolution to a level close to the diffusion limit resolution (Siems et al., 1994; Wu et al., 1998; Revercomb and Mason, 1975). This resolution refers to the highest resolution that can be obtained by the instrument when the rest of the factors, such as the Coulomb repulsion between ions and the inhomogeneity of the electric field, are neglected and only the effect caused by diffusion is considered. To further improve the separation capacity of IMS, this limit needs to be broken from the principle.

In this work, an inverse diffusion counterbalance method is proposed. The strategy is similar to the stimulated emission depletion (STED) method, which uses a ring of depleted light to counteract diffraction effects of the excited light. In our method, an inverse diffusion of a dip in the ion beam is used to counteract the diffusion of the pulsed ion packet. The counteracting process can be implemented in three steps: the first step is to create a normal pulsed ion peak; the second step is to create an inverse blank peak (Tabrizchi and Jazan, 2009) and subtract the baseline; and the third step is to subtract the results obtained from the first and second steps. The exact value of the method in terms of resolution improvement ratio was obtained mathematically, and we verified this conclusion by finite element simulation. The effect of different *w*_i_ and diffusion coefficients on the degree of implementation of this method was also examined.

## 2 Method

### 2.1 Implementation steps

In the field of optical microscopy, the smaller the area of the excitation spot, the higher the resolution of the image. However, the area of the excitation spot has a minimum value because of the diffraction of light. Stefan W. Hell and Jan Wichmann (Hell and Wichmann, 1994) use ring light with quenching effect to counteract the excitation effect at the edge of the excitation spot. This results in a reduction of the actual excitation area and breaks the diffraction resolution limit. The method is called the stimulated-emission-depletion (STED). In the pulsed IMS, ion packet is put into the drift region in the form of pulses. The narrower the width of the ion packet, the higher the resolution. However, during the drift, the pulsed ion packet of the gas phase will diffuse towards the surrounding air, and the diffusion effect leads to an increase in width. In order to reduce the width, we adopt the strategy of STED, hoping to use diffusion in the opposite direction to counteract the diffusion of pulsed ion packet. For the inverse IMS (Tabrizchi and Jazan, 2009), the diffusion is in the opposite direction, which happens to be used to counteract it. By using the appropriate steps, this counteracting strategy can be applied in IMS.

**FIGURE 1:**
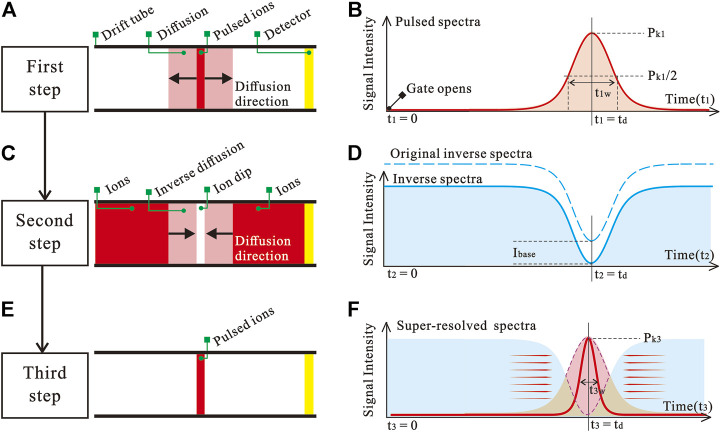
The steps of the inverse diffusion counterbalance method implementation. **(A)** Drift process of pulsed ion packet. **(B)** Schematic spectrum of a pulsed ion packet. **(C)** Drift process of inverse dip in ion beam. **(D)** Schematic inverse spectrum. **(E)** Pulsed ion packet after counterbalance. **(F)** Schematic spectrum that breaks the resolution limits.

The inverse diffusion counterbalance method can be accomplished through three steps. The first step is to generate a normal spectrum by putting ions in the form of a pulse into the drift tube. The pulsed ion packet diffuses from its center to the edge during the drift, and the process is shown in Figure 1A. Such diffusion leads to an increase in the width of the ion packet, which is the target we want to counteract afterwards. Figure 1B shows a schematic spectrum of the pulsed ion packet. The second step is to generate an inverse spectrum by a blocking pulse, as shown in Figure 1C. The ion dip generated in the second step has the same drift velocity and opposite diffusion direction (from the edge to the center) compared to the ion packet generated in the first step. Thus, the inverse spectrum can be used to counteract the diffusion part of the pulsed spectrum. In order to unify the minimum values of the spectrum obtained in the first and second steps, we subtracted a base parameter *I*_base_ from the inverse spectrum. The inverse spectrum is corrected to a minimum value of zero. The dashed and solid lines in Figure 1D show the original and corrected schematic spectra generated in the second step, respectively. The third step is to subtract the corrected inverse spectrum from the pulsed spectrum generated in the first step. For the pulsed spectrum, since what needs to be subtracted is only the intensity due to diffusion, the negative value in the spectrum after being subtracted should be set to zero. The equivalent drift process for this step is shown in Figure 1E, and the schematic spectrum is shown in Figure 1F. The area contained by the purple dashed line in Figure 1F is the remaining ion intensity after the diffusion has been counteracted.

In the actual experiment, the realization of this method relies mainly on the control of an ion gate. Ion gate generally consists of two sets of metal wires that control the opening and closing of the ion beam by the voltage difference between the wires (*V*_GVD_. When *V*_GVD_ is greater than the closing voltage (*V*_Closing_), the ions are pulled by the transverse electric field force and consumed on the lower voltage wires, which can cause the ion beam not to pass. In order to obtain the pulsed ion packet in the first step of the inverse diffusion counterbalance method, the ion gate needs to be opened (*V*_GVD_ = 0 V) for a short time (*t*_w_) and then kept closed (*V*_GVD_ > *V*_Closing_). The inverse dip in ion beam in the second step requires the ion gate to remain open at all times after a short time of closing.

### 2.2 Theoretical enhancement ratio

Here, we wish to derive the diffusion limit resolution of the spectrum obtained with the inverse diffusion counterbalance method. Similar to the preconditions (St. Louis et al., 1990; Siems et al., 1994) used in deriving the diffusion limit resolution of pulsed IMS, the following assumptions are made: space charge effects can be negligible; the diffusion motion is a gradient of concentration only along the *x*-axis; the normal and the inverse ion diffusion are symmetric and independent of the ion gate; and the width of ion packet at time zero is negligible.

We divide the derivation process into three parts. First we derive the relationship between the ion current (*I*_1_) generated in the first step with time and drift distance based on Fick’s law. Then we derive the relationship between the ion current (*I*_2_) generated in the second step with time and drift distance based on the symmetry of the diffusion. Finally, we obtain the relationship between the ion current (*I*_3_) generated by the inverse diffusion counterbalance method with time and drift distance by subtracting the above two ion currents, and calculate resolution enhancement ratio of the method.

For pulsed ion packets formed in the first step, the one-dimensional concentration of ions *C*_1_ versus time *t*_1_ and distance *x*_1_ is (Crank, 1975; Watts and Wilders, 1992)

$$C_{1}=\frac{C_{0}}{{\left(4\pi Dt_{1}\right)}^{1/2}}\exp\left[\frac{-{\left(x_{1}-K{\text{E}}_{d}t_{1}\right)}^{2}}{4Dt_{1}}\right],$$ (1)

where *E*_d_ is a constant electric field along the drift region, *C*_0_ is the one-dimensional concentration of the ion packet at time zero, *D* is the diffusion coefficient, and *K* is the ion mobility. Eq. 1 mainly describes the relationship between the one-dimensional concentration (*C*_1_) at different position (*x*_1_) on the ion packet and time (*t*_1_) when the ion packet moves along the *x*-axis. The ion concentration is an expression of the spatial distribution and cannot be used directly in the calculation of ion spectra. Therefore, we need to use the drift velocity of ions in an electric field to derive the ion concentration in the form of an ion current. Since the drift velocity *v*_d_ is lineraly related on *E*_d_, which is

$${\text{v}}_{d}=K\cdot {\text{E}}_{d}.$$ (2)

For a drift tube with a cross-sectional area of *S*, the current intensity of the received ions *I*_1_ is

$$I_{1}=C_{1}S{\text{v}}_{d}=C_{1}SKE_{d}=\frac{C_{0}SKE_{d}}{{\left(4\pi Dt_{1}\right)}^{1/2}}\exp\left[\frac{-{\left(x_{1}-KE_{d}t_{1}\right)}^{2}}{4Dt_{1}}\right].$$ (3)

So far, the relationship between the ion current generated in the first step (ion packet) versus time and drift distance is obtained.

In the second step, the current generated by the continuous ions is

$$I_{0}=C_{0}SKE_{d}.$$ (4)

Due to the symmetry of diffusion, the inverse ion current *I*_2_ with the base parameter subtracted versus time *t*_2_ and distance *x*_2_ is

$$I_{2}=I_{0}-I_{1}\left(t_{2},x_{2}\right)-I_{\text{base}},$$ (5)

where *I*_base_ is the base parameter to ensure that the minimum value of the inverse spectrum is zero. Suppose the peak height of the pulsed spectrum obtained in the first step is *I*_1pk_, then *I*_base_ satisfies that,

$$I_{\text{base}}=I_{0}-I_{\mathrm{1pk}}.$$ (6)

According to Eqs 3–6, it can be obtained that

$$I_{2}=I_{\mathrm{1pk}}-\frac{C_{0}SKE_{d}}{{\left(4\pi Dt_{2}\right)}^{1/2}}\exp\left[\frac{-{\left(x_{2}-KE_{d}t_{2}\right)}^{2}}{4Dt_{2}}\right].$$ (7)

So far, the relationship between the ion current generated in the second step (inverse dip in ion beam) versus time and drift distance is obtained.

In the third step, the initial time of the pulsed spectrum and the inverse spectrum is aligned. The variables of time *t*_3_ and distance *x*_3_ are the same as the first two steps, which is *t*_3_ = *t*_1_ = *t*_2_, *x*_3_ = *x*_1_ = *x*_2_. The expression for the ion current *I*_3_ of the spectrum is

$$\begin{array}{cc} I_{3} & =I_{1}\left(t_{3},x_{3}\right)-I_{2}\left(t_{3},x_{3}\right)\\ & =\frac{2C_{0}SKE_{d}}{{\left(4\pi Dt_{3}\right)}^{1/2}}\exp\left[\frac{-{\left(x_{3}-KE_{d}t_{3}\right)}^{2}}{4Dt_{3}}\right]-I_{\text{pk}1}. \end{array}$$ (8)

t is noted here that there is a possibility that *I*_3_ is negative in Eq. 8. According to the definition of our method, all negative values should be set to zero. Since the *I*_3_ used in the subsequent calculations are all positive values, that why we do not restrict *I*_3_ ≥ 0 here. For a drift region of length *L*, the magnitude of the peak happens at *t*_d_, where

$$t_{d}=L/KE_{d}.$$ (9)

According to Eq. 3, the peaks of the spectrum *I*_3pk_ is

$$I_{\mathrm{3pk}}=I_{\mathrm{1pk}}=I_{1}\left(t_{1}=t_{d},x_{1}=L\right)=\frac{C_{0}SKE_{d}}{{\left(4\pi Dt_{d}\right)}^{1/2}}.$$ (10)

According to the concept of full width at half maxima, Eq. 8, and Eq. 10, when *t*_3_ = *t*_d_ - *t*_w3_/2, the current intensity at position *x*_3_ = *L* is

$$\begin{array}{c} \frac{2C_{0}SKE_{d}}{{\left(4\pi Dt_{d}-t_{w3}/2\right)}^{1/2}}\exp\left[\frac{-{\left(L-KE_{d}\left(t_{d}-t_{w3}/2\right)\right)}^{2}}{4D\left(t_{d}-t_{w3}/2\right)}\right]-\frac{C_{0}SKE_{d}}{{\left(4\pi {\text{Dt}}_{d}\right)}^{1/2}}=I_{\mathrm{3pk}}/2=\frac{C_{0}SKE_{d}}{2{\left(4\pi {\text{Dt}}_{d}\right)}^{1/2}}, \end{array}$$ (11)

which yields:

$$\frac{3}{4t_{w3}^{1/2}}=\frac{1}{{\left(t_{d}-t_{w3}/2\right)}^{1/2}}\exp\left[\frac{-{\left(KE_{d}t_{w3}/2\right)}^{2}}{4D\left(t_{d}-t_{w3}/2\right)}\right].$$ (12)

Since the drift time (*t*_d_) is usually about 20 ms and the opening time (*t*_w_) is usually less than 0.2 ms, it can be assumed that *t*_d_ is much larger than *t*_w_. For *t*_d_ ≫ *t*_w_, (*t*_d_ − *t*_w3/2_) = *t*_d_. The resolution *R*_3_ of the inverse diffusion counterbalance method is

$$R_{3}=\frac{t_{d}}{t_{w3}}={\left[\frac{K^{2}{E_{d}}^{2}t_{d}}{16Dln\left(4/3\right)}\right]}^{1/2}.$$ (13)

Since the diffusion coefficient and the mobility are determined by the Nernst-Einstein equation:

$$D=Kk_{B}T/q,$$ (14)

where *k*_B_ is Boltzmann’s constant, *T* is the absolute temperature, *q* is the electrical charge of a particle. According to Eqs 9, 13, 14, the resolution *R*_3_ is

$$R_{3}={\left[\frac{E_{d}\text{L}\text{q}}{16k_{B}Tln\left(4/3\right)}\right]}^{1/2}.$$ (15)

Under weak electric field conditions, the diffusion limit resolution (Cohen and Karasek, 1970; Watts and Wilders, 1992) of traditional pulsed IMS can be written as

$${\text{R}}_{d}={\left[\frac{E_{d}\text{L}\text{q}}{16{\text{k}}_{B}Tln2}\right]}^{1/2}.$$ (16)

According to Eq. 15 and Eq. 16, the resolution enhancement ratio is

$$\alpha =\frac{R_{3}}{R_{d}}>1.55.$$ (17)

## 3 Experimental

**FIGURE 2:**
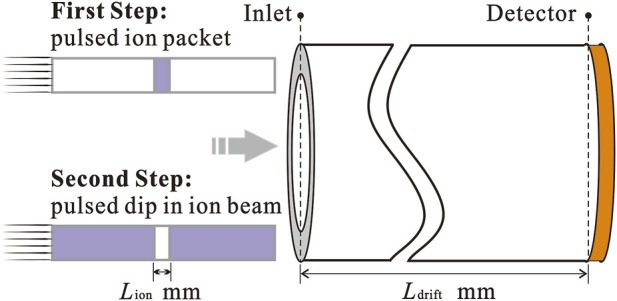
Schematic indicating the parameters of the drift tube.

A model is built in the simulation environment using the finite element method, as shown in Figure 2. The entrance is at *x* = 0 mm and the detector is at *x* = *L*_drift_. In order to divide the mesh in a more detailed way with limited computational power, the model is simplified in one-dimensional. A uniformly divided grid with a length of 5 µm is used. The cross-sectional area of the drift tube can be any value.

The drift electric field was set to 50 V/mm with a high potential at the ion inlet and a low potential at the detector position. In the first step, the function of the ion gate is achieved by putting an ion sequence of blank-ion-blank into the drift tube in temporal order. In the second step, the ion sequence of the inverse spectrum is ion-blank-ion. The blank is the absence of ions. The width of the ion packet in the first step and the width of the blank in the second step are guaranteed to be same, denoted by *L*_ion_. The initial moment of the spectrum is when the center of *L*_ion_ reaches *x* = 0 mm.

## 4 Results and discussion

**FIGURE 3:**
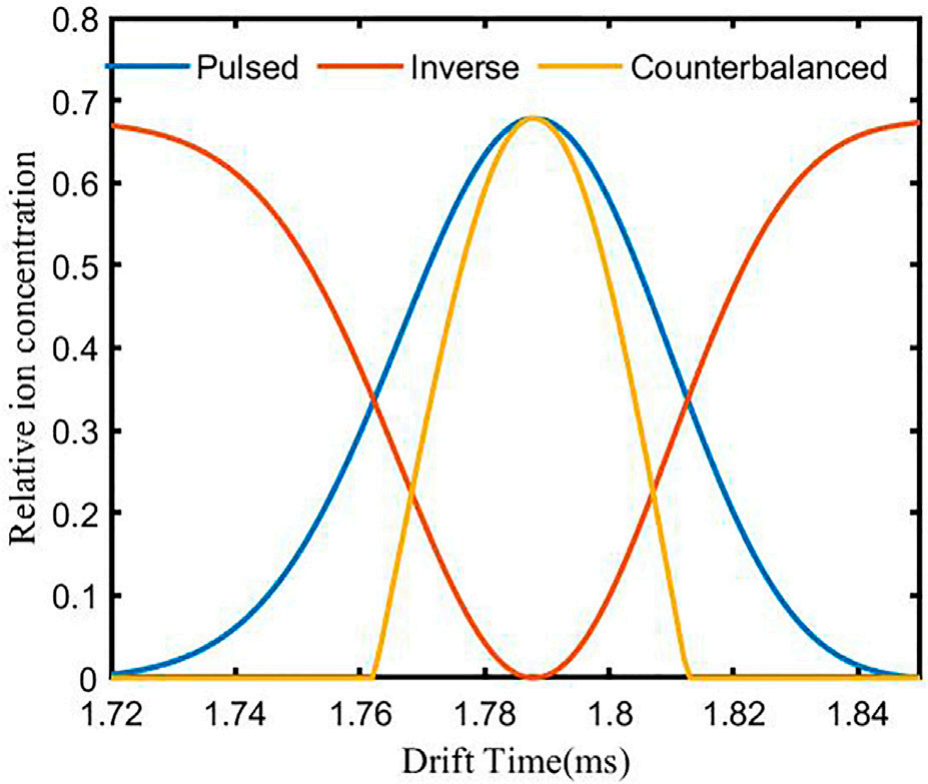
Simulation spectrum of a single type of ion using the inverse diffusion counterbalance method.

When *L*_drift_ = 10 mm and *L*_ion_ = 0.1 mm, the simulation spectrum of a single type of ion (*D* = 2.8⋅ 10^−6^ m^2^/s) is shown in Figure 3. The blue and the red curve are the pulsed and the inverse spectra generated in the first and second steps. The yellow curve is the counteracted spectrum obtained using the inverse diffusion counterbalance method. The drift time of the ion is 1.79 ms, and the resolution of the pulsed and the counteracted spectrum are 40 and 62, respectively. The resolution enhancement ratio *α*_r_ is 1.55, which is consistent with the theoretically calculated result.

**FIGURE 4:**
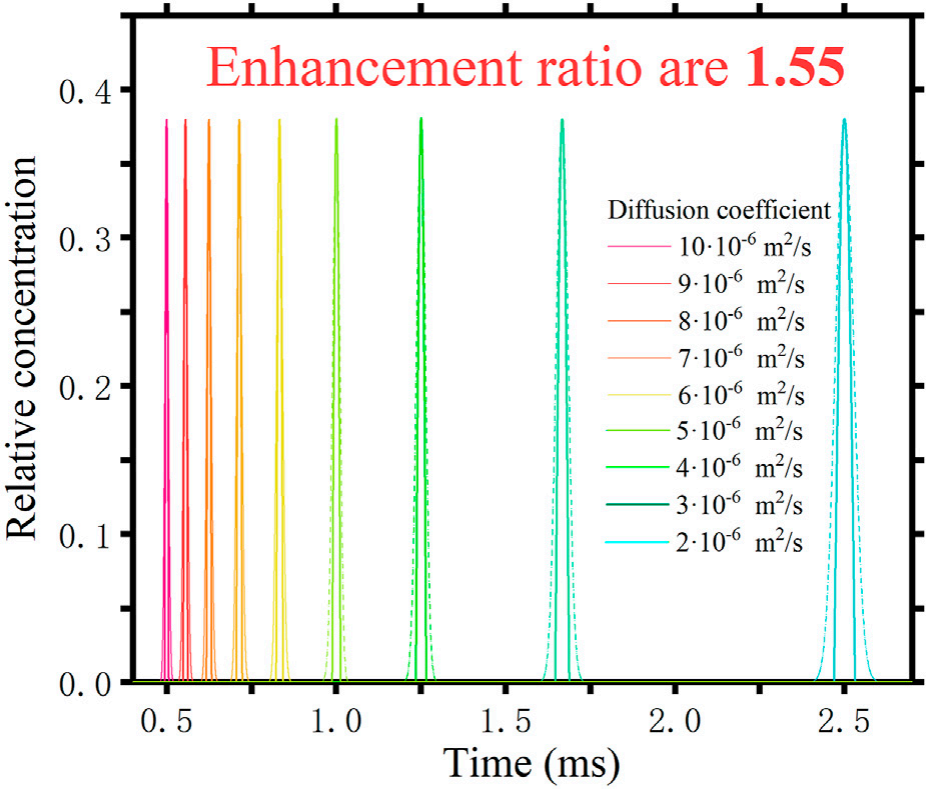
Spectra obtained using two methods for ions with different diffusion coefficients.

It is clear from the mathematical derivation that the beneficial effect of this method is independent of the diffusion coefficient of the ions themselves. We have also verified this in our simulations. Different species of ions (different *D*) are fed separately into the drift tube. Figure 4 shows the results. The dashed curve is the spectrum obtained using the traditional pulsed method and the solid line is the spectrum obtained using the inverse diffusion counterbalance method. The different colours correspond to the different *D*. It can be seen that ions with different diffusion coefficients arrive at the detection location at different times. We have tested nine different substances with diffusion coefficients ranging from 2 ⋅ 10^−6^ m^2^/s to 10 ⋅ 10^−6^ m^2^/s. They all have an resolution enhancement ratio of 1.55. Consistent with the theoretical derivation, the method does not have a differentiating effect depending on the diffusion coefficient of the sample.

**FIGURE 5:**
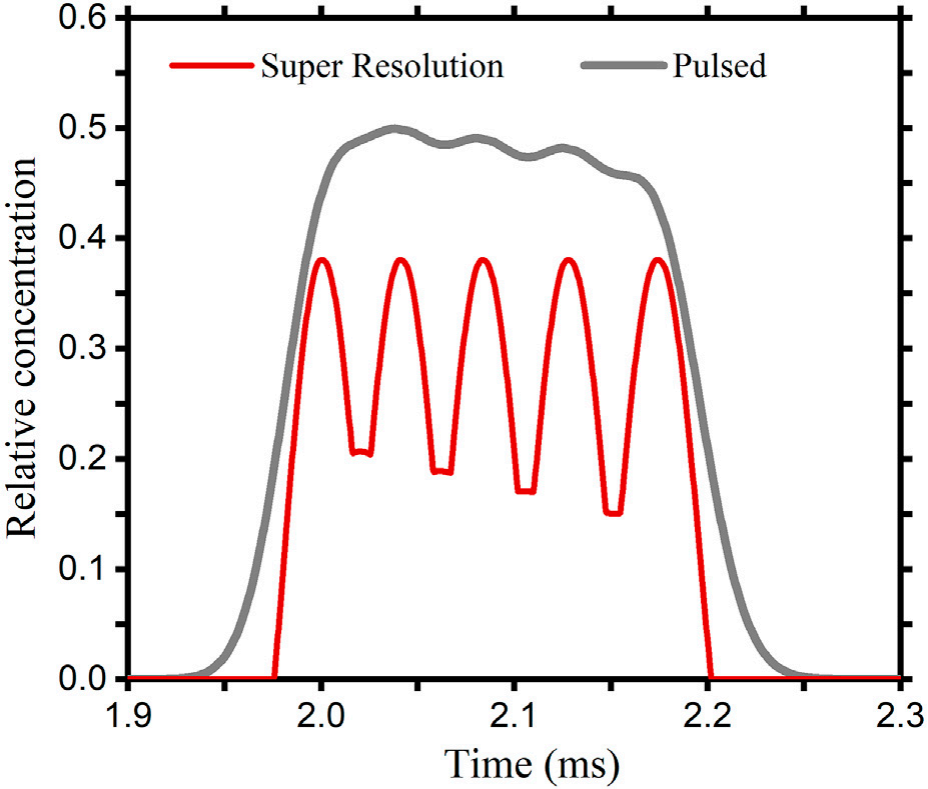
Results obtained by the pulsed method and the inverse diffusion counterbalance method, when ions with close diffusion coefficients are sampled simultaneously.

When ions with different diffusion coefficients are sampled simultaneously, ions with close diffusion coefficients will form overlapping peaks. The inverse diffusion counterbalance method is supposed to have a better separation. In the simulation model, we put in five different ion packets at the same time. The values of the diffusion coefficients are from 2.30⋅ 10^−6^ m^2^/s to 2.50⋅ 10^−6^ m^2^/s in intervals of 0.05⋅ 10^−6^ m^2^/s. Results are shown in Figure 5. The gray curve is the spectrum obtained by using the conventional pulsed method. There is little valid information in the result due to the mutual overlap between the peaks. The red curve is obtained by using the inverse diffusion counterbalance method. Five peaks with different drift times have been presented. To achieve the same level of resolution as the inverse diffusion counterbalance method, the drift tube needs to be extended to more than 2.4 times the original length using the conventional method.

The equivalent open gate time for these results are 17.9 µs. Such a short opening time can be neglected compared to the total drift time. Under these conditions, the diffusion effect is the main cause of the resulting increase in half-peak width. Therefore, the method of reducing the half-peak width by counteracting diffusion can be extremely effective, showing the same result as the theoretical value. When the initial width of the ion packet increases, this situation will change.

**FIGURE 6:**
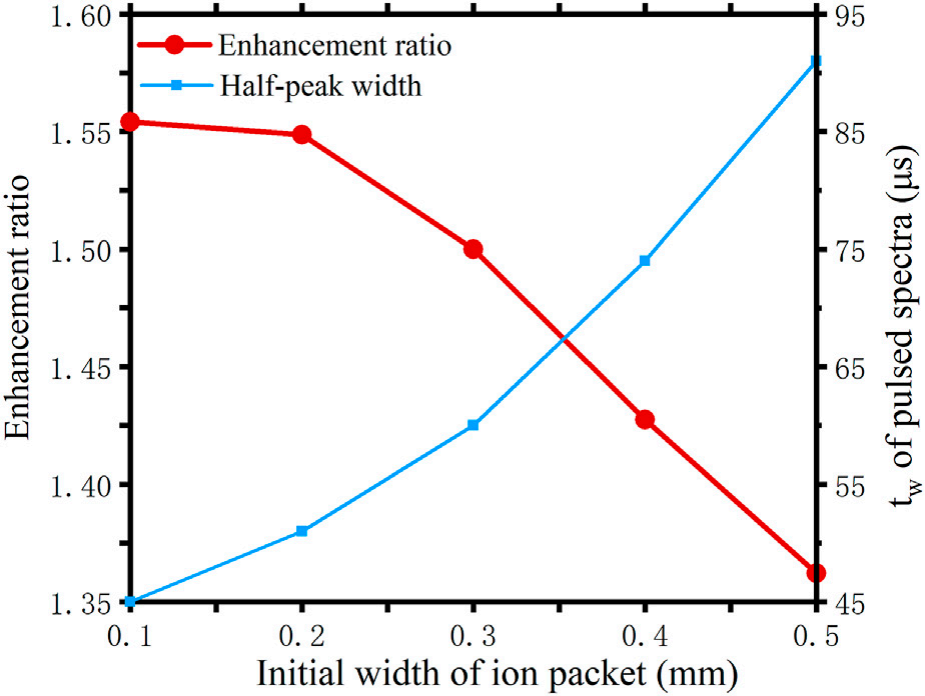
Effect of different initial width on the inverse diffusion counterbalance method.

The red curve in Figure 6 shows the variation of the enhancement when the initial width of the ion packet is increased. The blue curve shows the variation of the half-peak width of the pulsed spectrum. It can be seen that the wider the initial width of the ion packet, the less effective the method. This is easily understood because the method only works to counteract the effects caused by the diffusion. The larger the initial width as a percentage of the total ion packet width, the smaller the percentage of the diffusion width will be. Nevertheless, since the diffusion is inevitable, the method is also certain to result in an improvement in resolution.

## 5 Conclusion

For drift tube IMS, different ion species are separated by their drift time, and the ability to separate them is characterized by resolution. Due to diffusion, the maximum resolution that an IMS system can achieve is the diffusion limit resolution. This limit constrains the analytical capability of IMS. In the current work, an inverse diffusion counterbalance method has been proposed to break the limit. Through theoretical analysis and mathematical calculation, the resolution obtained with this method can be 1.55 times better than the conventional pulsed method. Since the method cannot counteract the effect of the initial width of the ion packet, the larger the initial width, the less effective the method will be. For ions with different mobility, this method has the same effect on resolution enhancement.

In order to verify the effectiveness of this method, a simplified 1D model was built in a finite element simulation software. The simulation results are in general agreement with the calculation. This method is a simple but very effective way of increasing resolution, and breaks the diffusion limit resolution. In the practical application of IMS, even if the hardware improvements have taken the instrument to its diffusion resolution limit, the resolution can still be improved to 1.55 times by using this method. The strategy of ion counterbalance may be applied to more broad-spectrum analytical instruments by experimental verification.

## Data Availability

The raw data supporting the conclusion of this article will be made available by the authors, without undue reservation.
